# Valorization of Industrial Lignin as Biobased Carbon Source in Fire Retardant System for Polyamide 11 Blends

**DOI:** 10.3390/polym11010180

**Published:** 2019-01-21

**Authors:** Neeraj Mandlekar, Aurélie Cayla, François Rault, Stéphane Giraud, Fabien Salaün, Jinping Guan

**Affiliations:** 1 Politecnico di Torino, Dept. of Applied Science and Technology, 15121 Alessandria, Italy; neeraj-kumar.mandlek@ensait.fr; 2 ENSAIT, GEMTEX-Laboratoire de Génie et Matériaux Textiles, F-59000 Lille, France; aurelie.cayla@ensait.fr (A.C.); francois.rault@ensait.fr (F.R.); fabien.salaun@ensait.fr (F.S.); 3 College of Textile and Clothing Engineering, Soochow University, Suzhou 215123, China; guanjinping@suda.edu.cn

**Keywords:** industrial lignin, Polyamide 11, phosphinate, thermal decomposition, fire retardancy

## Abstract

In this study, two different types of industrial lignin (i.e., lignosulphonate lignin (LL) and kraft lignin (DL)) were exploited as charring agents with phosphorus-based flame retardants for polyamide 11 (PA11). The effect of lignins on the thermal stability and fire behavior of PA11 combined with phosphinate additives (namely, aluminum phosphinate (AlP) and zinc phosphinate (ZnP)) has been studied by thermogravimetric analysis (TGA), UL 94 vertical flame spread, and cone calorimetry tests. Various blends of flame retarded PA11 were prepared by melt process using a twin-screw extruder. Thermogravimetric analyses showed that the LL containing ternary blends are able to provide higher thermal stability, as well as a developed char residue. The decomposition of the phosphinates led to the formation of phosphate compounds in the condensed phase, which promotes the formation of a stable char. Flammability tests showed that LL/ZnP ternary blends were able to achieve self-extinction and V-1 classification; the other formulations showed a strong melt dripping and higher burning. In addition to this, cone calorimetry results showed that the most enhanced behavior was found when 10 wt % of LL and AlP were combined, which strongly reduced PHRR (−74%) and THR (−22%), due to the interaction between LL and AlP, which not only promotes char formation but also confers the stability to char in the condensed phase.

## 1. Introduction

Over the past two decades, the sustainable development of flame retardant material has become a major topic of concern in industry and academia, due to the toxicity associated with flame retardants and increasing environmental and health safety regulations. This issue has driven the development of flame retardant systems made from renewable resources as possible alternatives to the non-halogenated ones [1]. Nowadays, Polyamide 11 (PA11), a renewable natural high-performance polymer derived from castor seed oil, has gained much attention because of its high mechanical strength and chemical resistance properties [2]. PA11 has been exploited in automotive, aerospace, sports applications, and textile industries [3,4,5]. However, low flame-retardant properties and extended dripping of PA11 limits its potential applications in high-performance textiles. In order to confer flame retardancy to PA11, intumescent systems based on bioresources are possible substitutes to halogen-free flame retardants. It is believed that the use of materials based on renewable resources can reduce the carbon footprint in our environment. Lignin consists of non-toxic polyaromatic polyols, one of the most abundant biopolymers. The majority of industrial lignin is produced annually by the pulp and papermaking industries and the lignocellulosic ethanol industries; approximately 70 million tonnes of lignins are produced per year worldwide [6,7]. However, around 2% is isolated and comprehensively utilized, and the rest is primarily incinerated for energy recovery. In recent years, the valorization of lignin compounds has attracted growing interest for its potential applications in polymers, due to its competitive price, abundant availability, reactive functional groups, high aromatic carbon content, and tailored capability for structural modification [7,8,9,10].

In particular, lignin has been employed to improve the thermal stability of thermoplastic polymers [11,12,13], exploiting the thermal decomposition of lignin, which takes place over a broad temperature range, since various aromatic functional groups have different thermal stability. More recently, lignin has shown great potential as a biobased carbon source in an intumescent system with traditional flame retardants [14,15] because of its high char yield (about 50–60 wt %) upon thermal decomposition in inert atmosphere [16]. The char-formation can reduce the heat release rate of the polymeric material during the combustion process. Furthermore, char forming ability of lignin can be exploited for the design of environmentally friendly intumescent systems with phosphorus-based non-halogenated flame retardants. In this context, among various phosphorus flame retardants, metal phosphinates are considered very efficient and have several advantages, being mostly non-hygroscopic, non-toxic, thermally stable (>350 °C), and resistant to hydrolysis. In particular, AlP and ZnP are the most widely used and are found to be very effective flame retardants, especially for polyesters and polyamides [17]. However, they show satisfactory flame-retardant properties only on rather a high loading of about 30 wt % [18]. Phosphinate loading was conceivably reduced in the presence of synergistic or catalytic compounds. Furthermore, it was found that some nitrogen-containing compounds such as melamine polyphosphate (MPP) and melamine cyanurate (MC) show synergism when combined with phosphinate [19,20]. The efficiency was further improved by the addition of zinc borate (ZnB) to AlP and MPP system [21]. The presence of ZnB led to the formation of stable residue and a dominant barrier effect. In another study, Didane et al. exploited the synergistic effects of Polyhedral Oligomeric Silsesquioxane (POSS) with ZnP in developing flame retardant PET fibres [22]. The addition of synergistic agents enhances the ZnP efficiency, by reducing its required loading in the polymer matrix. 

In order to promote the utilization of biobased lignin as flame retardant additive, our previous study has elucidated the use of lignin (pure grade) as a carbon source with ZnP [23]. Lignin used in this work was low sulfonate containing (4% sulphur) alkali kraft lignin. Different formulations of pure lignin and ZnP were prepared and their fire retardancy effect and thermal decomposition behaviour were investigated. It was found that the blends containing 10 wt % of both additives showed most enhanced flame-retardant properties, due to the formation of a stable char layer with barrier features. Besides, in the present work, we have focused on the use of chemically different industrial lignin (i.e., kraft lignin and lignosulfonate lignin) combined with two different phosphinates, i.e., aluminum phosphinate (AlP) and zinc phosphinate (ZnP). Lignins exploited in this work were chemically different and less pure than the same used in the previous study. The purpose of this study is to compare the flame-retardant effectiveness of industrial lignin with phosphinate in PA11. To this aim, the different formulations based on phosphinates and lignins were prepared by melt extrusion and thoroughly investigated. The morphology of prepared blends examined by Scanning Electron Microscopy (SEM) analyses. Their effects on the thermal stability and char-forming ability of blends were thoroughly investigated by thermogravimetric (TG) experiments. Fire behaviour of prepared blends was assessed by the vertical flame spread and cone calorimetry tests. Furthermore, the morphology of char residues after cone calorimetry tests was also investigated by SEM.

## 2. Experimental

### 2.1. Materials and Processing

A biobased Polyamide 11 (PA11), Rilsan^®^ BMNO-TLD; *M*_n_ = 17,000 g/mol, melt flow index (MFI) = 14–20 g/10 min at 235 °C, supplied from Arkema ( Colombes, France), was chosen as the polymer matrix. Flame retardants zinc phosphinate (ZnP), Exolit 950 and aluminum phosphinate (AlP), Exolit 1230, were supplied by Clariant, Muttenz, Switzerland. Two different types of industrial lignins were used as a carbon source, varying in chemical nature: the first one is a lignosulphonate lignin (coded as LL), provided from Domsjö Fabriker AB ( Örnsköldsvik Sweden) and the second one is an alkali kraft lignin (coded as DL) obtained from UPM Biochemicals, Helsinki, Finland (European distributor of Domtar BioChoice^®^ lignin). LL contains mainly Na-lignosulfonate (about 70%) and small amounts of Mg and Ca lignosulfonates, and some impurities such as ash and carbohydrates (about 20%). DL comprises mostly alkali kraft with 90% purity level. All the materials were dried at 80 °C for 12 h before use.

### 2.2. Blends Preparation

The different blend formulations based on PA11, lignins (LL or DL) and phosphinates (ZnP or AlP) were prepared by melt extrusion and labelled as PA_X_-L_Y_-P_Z_, where X represents the amount of PA11, L the type of lignin used, Y the wt % lignin loading, P the type of phosphinate, and Z the wt % phosphinate content. A total of 20 wt % loading was used in blend formulation; phosphinate, being the major component, was varied from 10 to 15 wt % and lignin from 5 to 10 wt % (Table 1). Blends were prepared using a co-rotating intermeshing twin-screw extruder (Thermo Haake, screw diameter = 16 mm, L/D = 25); the temperatures of the five heating zones ranged from 170 to 220 °C, and the rotation speed was set at 100 rpm. In all cases, the extruded rods were pelletized for thermal analysis. 3 mm thick plates were manufactured by compression molding using a Collins Teach Line 200 hydraulic press, operating at 60 bars and 220 °C for 3 min. Specimens in accordance with cone calorimeter (100 × 100 mm²) and UL-94 flammability (125 × 12.5 mm^2^) tests were prepared and used.

### 2.3. Morphology

Morphology and distribution of the fillers in polymer matrix were assessed by SEM; analyses were carried out using a LEO-1450VP apparatus (Carl Zeiss, Oberkochen, Germany), equipped with a X-ray probe (INCAEnergy Oxford Instruments, Cu Kα X-ray source, k = 1.540 562 Å, Abingdon-on-Thames, UK) under the voltage of 20 kV. Energy dispersive X-rays (EDX) elemental mapping was also used for assessing the dispersion of phosphinate and lignin in PA11 matrix. Pellets of different blends were fractured in liquid nitrogen and then coated with a thin conductive gold layer.

### 2.4. Thermal Decomposition

Thermogravimetric (TG) analyses were carried out with a TA instruments thermal analyzer Q500 (New Castle, DE, USA), either under air or nitrogen atmosphere at a purge rate of 60 mL/min, with a heating rate of 10 °C/min from 50 to 800 °C, using alumina pans and sample weight of 10 ± 0.2 mg. TG curves were recorded from experiments and dTG (derivative) curves were obtained from TA universal data analysis software for all the samples. The decomposition parameters, such as the temperature at 5% weight loss (*T*_5%_), and residue at 700 °C, were obtained from TG curve. Furthermore, the maximum mass loss rate (MMLR) and the corresponding temperature (*T*_max_) were obtained from dTG curves. During TG analyses in air, the main decomposition step (*T*_max1_) and the second thermo-oxidative degradation step (*T*_max2_) were evaluated.

The weight difference curves were plotted in order to determine the potential increase or decrease in the thermal stability of the blends. The weight difference curves were computed for the loaded samples, and correspond to the weight difference between the experimental and theoretical TG curves:

(1) $$\Delta (M(T))=M_{\exp}(T)-M_{theo}(T)$$

Where, Δ(M(T)) is a residual weight difference, $M_{\exp}(T)$ is the experimental residual weight of blends (variation with temperature *T*), $M_{theo}(T)$ is the theoretical residual weight of the same composition computed by a linear combination between the experimental weights of PA11 and additives. The curves show the subsequent interaction between additives and polyamide matrix by the observation of an increase or decrease of the thermal stability.

### 2.5. Fire Behavior

The flammability was evaluated on sample sheets (125 × 12.5 × 3 mm^3^) by vertical flame spread tests according to IEC 60695-11-10 [24], also known as UL 94 burning flame test, and used for plastic materials. This test is aimed at assessing the material capability to extinguish a flame. Materials were classified on the basis of burning rate, time to flame extinction, and dripping during burning.

Cone calorimeter tests were carried out at a heat flux of 35 kW m^−2^ to assess the forced combustion behavior of sheets (100 × 100 × 3 mm^3^) in accordance with ISO 5660 standard [25]. The distance between the sample and the heating cone was increased to 60 mm due to the material swelling. It was assumed that this swelling behavior was attributed to stress release under heat flux, which was formed during cone plate manufacturing. Before performing the tests, all specimens were conditioned at 23 °C and 50% RH for 72 h. Three tests were carried out on each formulation, and the results averaged. According to this analytical method, critical parameters were evaluated, namely: time to ignition (TTI), heat release rate (HRR), total heat release (THR); furthermore, parameters related to smoke release such as total smoke release (TSR), CO_2_, CO, and CO_2_/CO yield were also evaluated.

## 3. Results and Discussion

### 3.1. Morphology of Blends

**Figure 1:**
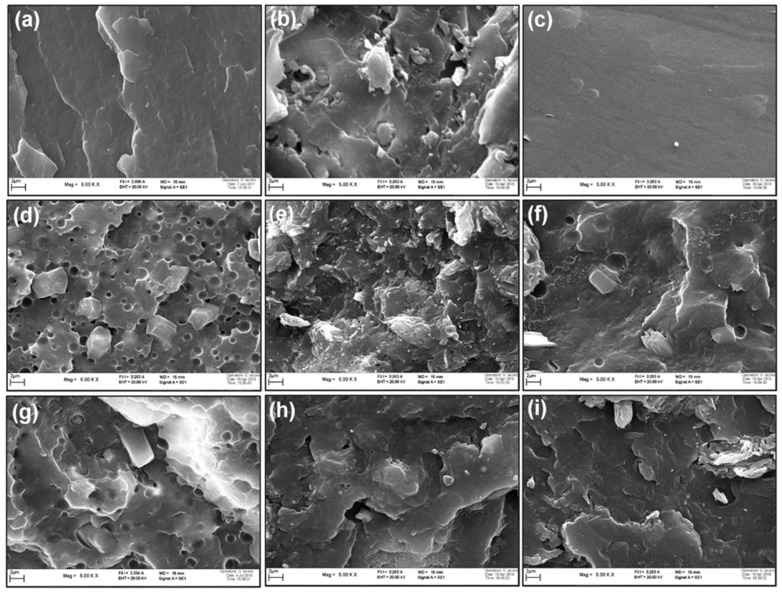
Scanning Electron Microscopy (SEM) micrograph of polyamide 11 (PA11) and its binary and ternary blends. (**a**) PA11; (**b**) PA_80_-LL_20_; (**c**) PA_80_-DL_20_; (**d**) PA_80_-ZnP_20_; (**e**) PA_80_-AlP_20_; (**f**) PA_80_-LL_10_-ZnP_10_; (**g**) PA_80_-DL_10_-ZnP_10_; (**h**) PA_80_-LL_10_-AlP_10_; (**i**) PA_80_-DL_10_-AlP_10_ at 5000× magnification.

The dispersion of the lignin and phosphinates in the PA11 matrix was assessed by SEM. The final properties of the blends, such as the flame-retardant performance, depend on the quality of dispersion of the fillers in the polymer matrix [26]. Figure 1a–i shows fractured cross-section images of PA11 and its binary and ternary blends with lignin and phosphinate. It was experienced that during thermo-mechanical compounding the additives size in the polymer matrix is reduced from their original size (Figure S1, Supporting information). From Figure 1b,c, the distribution of LL and DL can be observed for PA_80_-LL_20_ and PA_80_-DL_20_ binary blends, showing no aggregates, and particles are distributed in the entire surface. However, larger size (less than 10 µm) immiscible lignin particles can be observed due to a higher concentration of lignin in binary blends. In addition to this, PA_80_-ZnP_20_ binary blend exhibit distribution of ZnP particles in the polymer matrix. The larger size ZnP particles are observed to be due to broad size distribution in pristine ZnP. It is observed that blends containing ZnP show circular cavity, which can be ascribed to a fusible characteristic of ZnP [27]. However, a micrograph of the PA_80_-AlP_20_ binary blend (Figure 1d) shows homogenous distribution and good compatibility between AlP and polymer phase.

**Figure 2:**
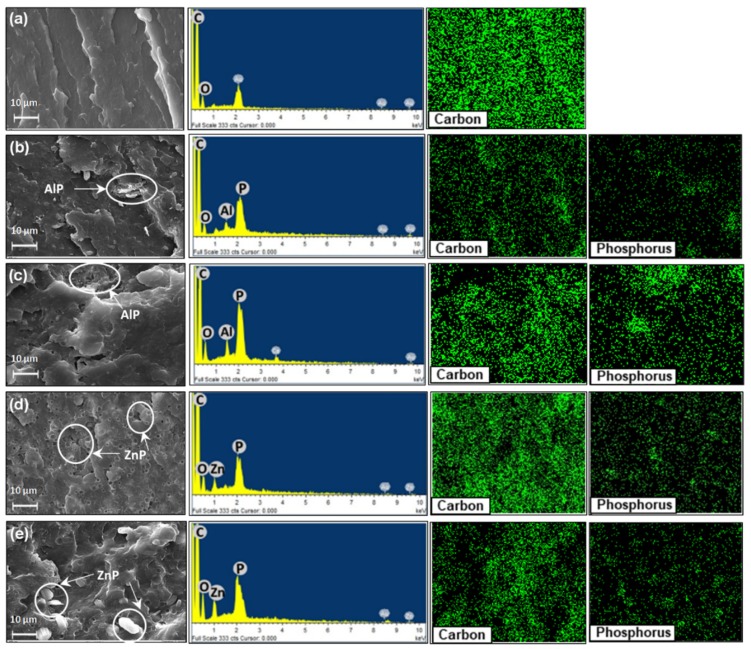
Scanning Electron Microscopy (SEM) micrographs of unfilled polyamide 11 (PA11) and the ternary blends. (**a**) PA11; (**b**) PA_80_-LL_10_-AlP_10_; (**c**) PA_80_-DL_10_-AlP_10_; (**d**) PA_80_-DL_10_-ZnP_10_ and (**e**) PA_80_-LL_10_-ZnP_10_ at 2500× magnification and corresponding energy dispersive X-rays (EDX) spectra and elemental maps.

In order to further assess the dispersion, EDX maps of Carbon and Phosphorus and EDX spectra of ternary blends containing 10 wt % loading of both the fillers are also presented in Figure 2a–e. In fact, it is commonly accepted that EDX mapping spectra can give the qualitative evidence of dispersion and distribution level of the elements [28]. As far as lignin distribution is concerned, it was observed during the blend preparation process that lignin particles are broken down and mixed well with PA11 matrix; furthermore, SEM micrographs show a homogeneous distribution of lignin particles in the polymer matrix, as no phase separation or agglomeration is observed; this indicates reasonable compatibility between lignin and PA11 [16]. In addition to this, Figure 2b,c shows the morphology and distribution of AlP in combination with LL and DL. AlP was found in the entire surface, though small agglomerations (<10 µm) are randomly observed. EDX mapping of phosphorus confirms the uniform distribution of the phosphinate in the blends. Carbon (C), Oxygen (O), Aluminium (Al) and Phosphorus (P) elements were mainly detected and confirmed the presence of AlP and lignin within the polymer matrix. In addition to this, the ternary blends of ZnP with DL/LL (Figure 2d,e) display a larger particle size of ZnP embedded in the matrix; this morphology was observed due to the broad range of size distribution in the pristine ZnP (Figure S1, Supporting information). EDX elemental mapping and corresponding spectra further confirm the identification of C, O, Zn, and P in the entire surface. However, apart from larger size, ZnP particles are uniformly dispersed in the entire surface, without agglomeration phenomena.

### 3.2. Decomposition Behavior

**Figure 3:**
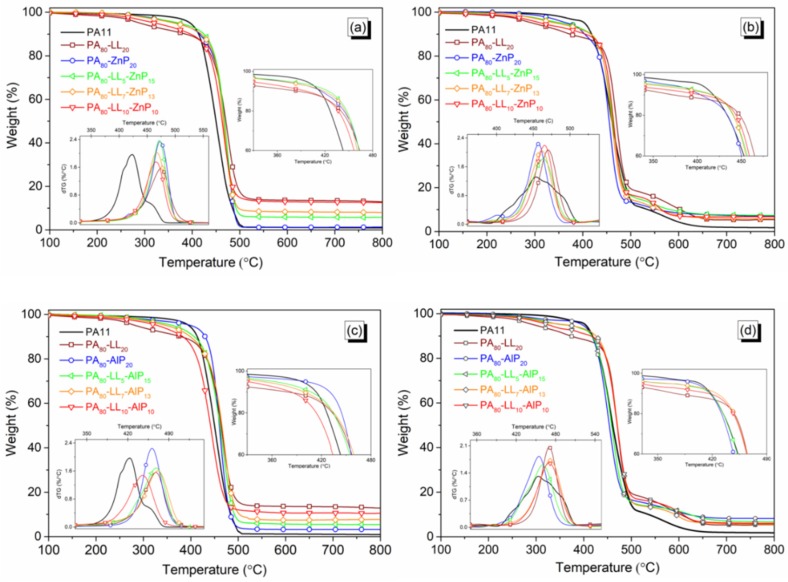
Thermogravimetric (TG) curves of polyamide 11 (PA11) blends of lignosulphonate lignin (LL) in combination with zinc phosphinate (ZnP) and aluminum phosphinate (AlP) in N_2_ ((**a**) and (**c**)) and air atmosphere ((**b**) and (**d**)).

**Figure 4:**
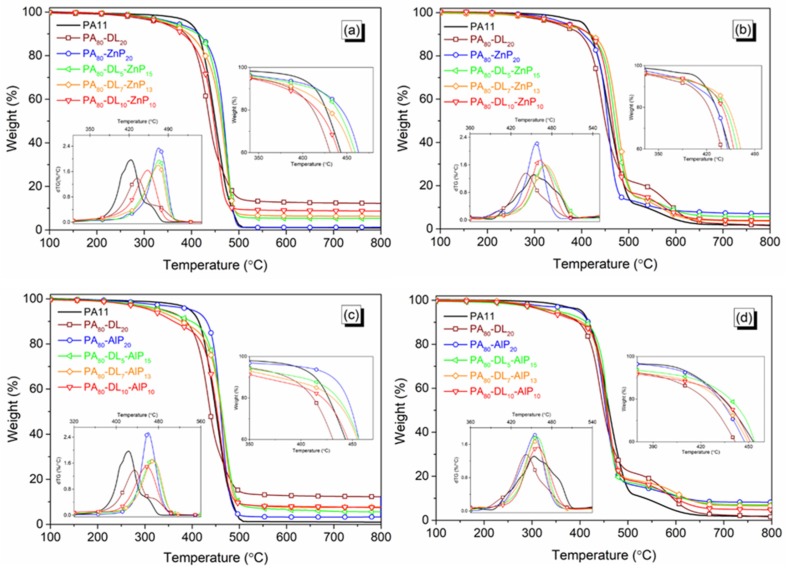
Thermogravimetric (TG) curves of polyamide 11 (PA11) blends of kraft lignin (DL) in combination with zinc phosphinate (ZnP) and aluminum phosphinate (AlP) in N_2_ ((**a**) and (**c**)) and air atmosphere ((**b**) and (**d**)).

The thermal and thermo-oxidative stability of PA11 and blends was assessed by TG analyses in N_2_ and air atmosphere, respectively. Thermogravimetric data are collected in Table 2. However, TGA curves of neat material are presented in Figure S2 (Supporting information). The thermal stability and degradation profile of the binary and the ternary blends are shown in Figure 3 and Figure 4. In N_2_ atmosphere, unfilled PA11 starts decomposing at 396 °C (*T*_5%_) and shows single decomposition step with a maximum mass loss at 430 °C (*T*_max_) without leaving any residue at the end of the test: a similar behavior was reported in previous studies [16,23]. However, in air, PA11 decomposes in two steps; the first step is at around 454 °C (*T*_max1_), giving rise to the formation of volatile products. The second step, at around 574 °C (*T*_max2_), can be attributed to the further oxidation of hydrocarbon species with the formation of CO and CO_2_, leading to a consistent weight loss until 800 °C. 

TGA data of the binary blends are summarized in Table S1 (Supporting information). The presence of LL or DL in binary blends initiate the degradation (i.e., lowering 5 wt % loss) at 285 and 341 °C, respectively. In nitrogen, LL shows a slower decomposition rate compared to DL, leading to the thermal stability shifts to a higher temperature; consequently, its blend generates a higher amount of char residue (13.5 wt % at 700 °C) as compared to the theoretical one (12.5 wt %). Furthermore, in inert atmosphere, the thermal stability of binary blends of ZnP and AlP increases, and a single decomposition step occurs within 460 to 470 °C. In addition, a slightly higher residue for PA_80_-AlP_20_ blend reveals that the interaction between AlP and polymer matrix, resulting in a thermally stable residue compared to the residue from the PA_80_-ZnP_20_ blend [29].

In air, the blends containing DL generate a lower amount of char residue because of the destabilization of the resulting char. Conversely, blends containing a higher amount of ZnP or AlP generate a higher residue, due to the formation of the phosphate compounds in the condensed phase, as it will be later demonstrated.

When phosphinates (ZnP/AlP) and lignins (LL/DL) were combined with PA11, the decomposition behaviour changed (Figure 3 and Figure 4). In all ternary blends, the presence of any lignin reduces the initial decomposition temperature (*T*_5%_); at the same time, *T*_max_ increases. This behaviour is ascribed to the lignin degradation, which starts at a lower temperature and continues at a very slow rate; at the end, a noticeable amount of char residue is collected. It is noteworthy that, increasing the LL loading in PA-LL-ZnP blends strongly influences the *T*_5%_ and MMLR; as a consequence, the recorded experimental char residue is higher with respect to the theoretical one, thus indicating the positive interaction within the additives. These blends generate slightly higher residue as compared to the lignin and ZnP combination used in our previous study [23]. It is assumed that, during the decomposition of LL, the sulfonate compounds release SO_2_ and transform it into thermally stable Na_2_SO_4_, giving rise to a stable char [30]. Unlike PA–LL–ZnP blends, increasing DL content in its ternary blends slightly influence the *T*_5%_ and the thermal stability of the ternary blends; in particular, a higher mass loss and a lower char residue at the end of the experiment are observed. The lower production of the char residue from DL containing blends with respect to LL counterpart can be attributed to their different chemical structure; in particular, DL contains a higher number of less thermally stable methoxy groups that give rise to the formation of more volatile products during decomposition [31]. As far as the thermo-oxidative stability is concerned, the ternary bends show a similar trend, with two decomposition steps: the first one is attributed to the main degradation occurring within 450 to 470 °C (*T*_max1_), giving rise to the formation of volatile products such as phosphinate compounds and phosphinic acid; the second step, occurring at around 550 to 610 °C (*T*_max2_) is attributed to the formation of thermally stable phosphate compounds in the condensed phase [32,33]. It was noticed that ternary blends containing AlP show a second decomposition step between 600 and 610 °C (*T*_max2_) compared to ZnP containing ternary blends, which give *T*_max2_ at lower temperatures (i.e., within 550 and 580 °C). This finding confirms that the phosphate compound formation from AlP is more favored than that from ZnP. Furthermore, the residue obtained in oxidative condition was lower compared to the theoretical one; this finding can be ascribed to the presence of an impurity in the lignin due to their industrial nature, which catalyzes a further oxidation with the formation of CO and CO_2_.

**Figure 5:**
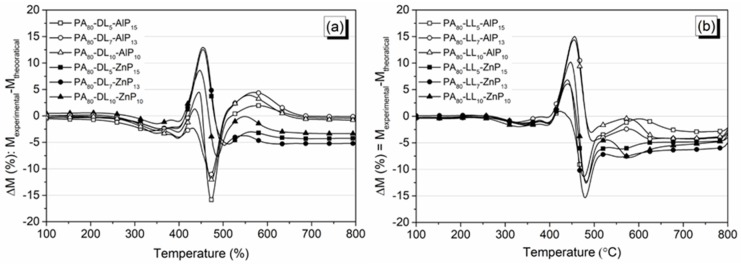
Curves of residual mass loss difference for polyamide 11 (PA11) ternary blends with lignosulphate lignin (LL) (**a**) and with kraft lignin (DL) (**b**) in air.

The thermo-oxidative behavior of the prepared blends was assessed through weight loss difference curves ∆M (%). Those collected in Figure 5a,b, show the interactions among lignin, phosphinates, and PA11. Figure 5a shows stabilization and destabilization region in ternary blends of LL with AlP and ZnP. The blends show slightly destabilization (∆M is below −2%) region between 270 and 380 °C and then stabilization from 380 to 460 °C; afterwards, the destabilization region continues until 800 °C. In addition, an increase of ∆M and thermal stability was observed with increasing LL loading in blends, hence confirming the positive interaction of LL with fire retardant additives. For example, PA_80_-LL_10_-AlP_10_ blend shows stabilization from 415 to 482 °C, and then destabilization of transient char continues up to 800 °C. On the other hand, the presence of DL with AlP and ZnP decreases the thermal stability, as assessed by the destabilization at a lower temperature (within 270 and 400 °C, Figure 5b). Further, PA–DL–AlP blends show small stabilization region (∆M is below 5%) between 400 and 450 °C and large destabilization (∆M is below −16%) between 450 and 520 °C due to the evolution of more volatile compounds. Finally, stabilization of transient char continues until 800 °C due to the formation of a stable phosphate layer in the solid phase. The replacement of AlP with ZnP widens the stabilization region (∆M is about 12%) between 400 and 500 °C; then, the destabilization (∆M is less than −5%) of transient char continues until 800 °C. Interestingly, increasing the DL content has no positive influence on mass difference and stabilization region, thus indicating a poor interaction of DL with fire retardant additive in PA11.

### 3.3. Flammability Behavior

**Figure 6:**
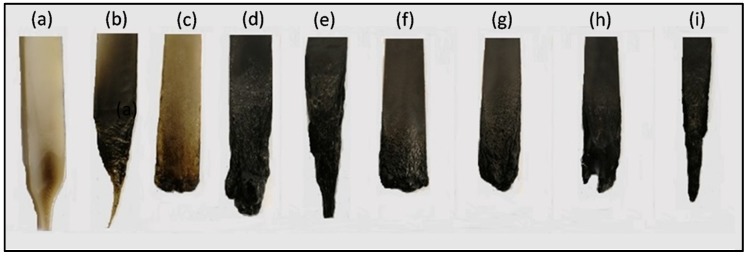
Pictures of PA11 blends specimen left after UL 94 vertical flame test. (**a**) PA11; (**b**)PA_80_-ZnP_20_; (**c**) PA_80_-AlP_20_; (**d**) PA_80_-LL_20_; (**e**) PA_80_-DL_20_; (**f**) PA_80_-LL_10_-ZnP_10_; (**g**) PA_80_-DL_10_-ZnP_10_; (**h**) PA_80_-DL_10_-ZnP_10_; (**i**) PA_80_-DL_10_-AlP_10_.

The results of the UL94 tests for PA11 and the ternary blends are summarized in Table 3, and the typical pictures of the specimens left after the tests are shown in Figure 6. However, the UL94 tests data for the binary blends are reported in Table S2 (Supporting information). Dripping was observed in all the blends, except PA_80_-AlP_20_, which improves the flame-retardant performance and achieves self-extinction (V-0 rating); further, no melt dripping is observed. This finding can be ascribed to the release of phosphinic acid and phosphinate compounds in the gas phase, which dilutes the fuel. Conversely, 20 wt % ZnP loading in PA11 is not sufficient to improve flame retardancy and UL94 rating; in fact, PA_80_–ZnP_20_ specimens lead to high flammability and dripping (Table S2, Supporting information).

In addition to this, the presence of LL in ternary blends decreases the total combustion time. However, the best flame-retardant results were obtained with PA–LL–ZnP blends, specifically with 7 and 10 wt % loading of LL (i.e., for PA_80_–LL_7_–ZnP_13_, PA_80_–LL_10_–ZnP_10_ blends), which achieve self-extinction and V-1 classification. Interestingly, PA_80_–LL_10_–ZnP_10_ shows the minimum ZnP loading. It is expected that the presence of sulfonate functionality in LL may lead to the formation of a thermally stable compound. In fact, increasing ZnP loadings deteriorate the performances, as ZnP promotes melt dripping phenomena [34]. As regards to the flame-retardant action, it was assumed that ZnP decomposition produces phosphinate compounds in the gas phase that can release P-O^•^ radicals: this latter can act as radical scavengers and lead to flame inhibition through radical trapping, hence improving the flame retardancy of blends [35,36]. These results demonstrate that the proposed LL/ZnP combinations are potentially effective. Conversely, the presence of DL in ternary blends does not show significant improvement under UL94 tests, as all these ternary blends have a V-2 classification. This behavior was attributed to the rapid decomposition of DL in air; further, DL decomposed mainly (*T*_max1_) during the decomposition of PA11.

### 3.4. Forced-Combustion Behavior

**Figure 7:**
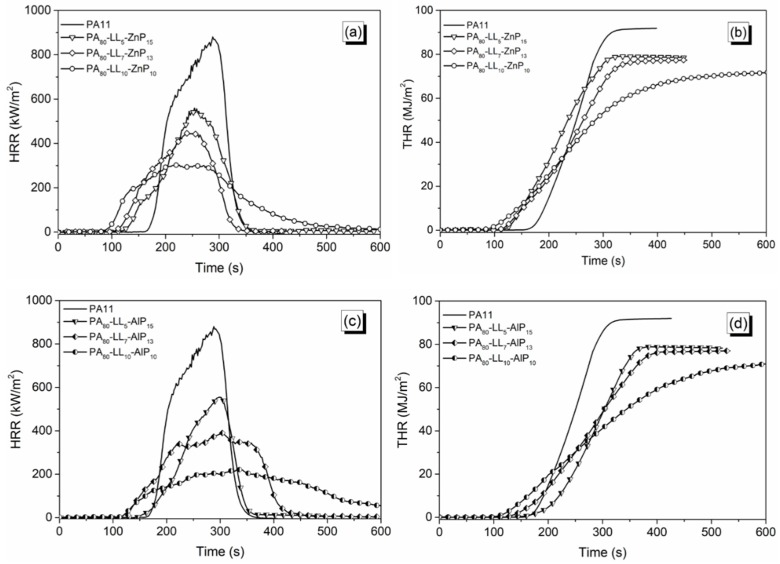
Heat release rate (HRR) and total heat release (THR) curves of polyamide 11 (PA11) blends. (**a**) and (**b**) for PA–LL–ZnP blends; (**c**) and (**d**) for PA–LL–AlP blends.

**Figure 8:**
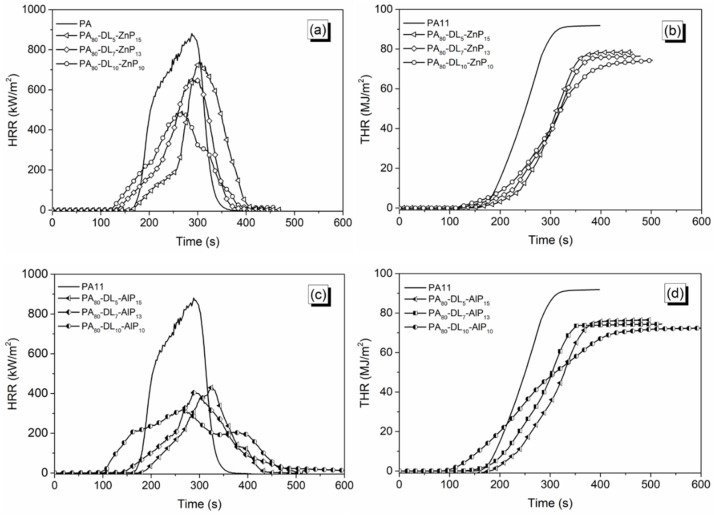
Heat release rate (HRR) and total heat release (THR) curves of polyamide 11 (PA11) blends. (**a**) and (**b**) for PA–DL–ZnP blends; (**c**) and (**d**) for PA–DL–AlP blends.

In order to simulate the fire hazards under a real fire scenario, cone calorimetry tests were performed using 35 kW/m² heat flux and 60 mm separation length. Since the distance from the sample surface to the spark igniter increases, the volatiles leaving from the heated sample and the oxygen from air have more time to mix before reaching the spark igniter. This is indicated by an increase in time to ignition. The heat release rate (HRR) and total heat release (THR) are potential fire parameters to evaluate the combustion behaviour of a material exposed to certain heat flux [37,38]. The HRR and THR curves are shown in Figure 7 and Figure 8 and the cone calorimetry data for PA11 and the ternary blends are collected in Table 4. However, heat release curves and the cone calorimetry data for the binary blends are presented in Figure S3 and Table S3 (Supporting information). Figure 7a–d shows the influence of LL with ZnP/AlP on HRR and THR. In particular, the ternary blends containing LL and ZnP showed lower TTI as compared to unfilled PA11, as anticipated by TG analyses in air: in fact, the adding of 5 to 10 wt % LL strongly reduces its *T*_5%_, promoting, at the same time, the formation of a thermally stable char residue. A similar trend was observed during combustion tests: TTI value decreases with increasing LL content from 5 to 10 wt % in blends; this finding is attributed to the rapid mass loss of LL taking place before the decomposition of PA11. Besides, PHRR and THR values substantially drop (by 64% and 22%, respectively) for PA_80_-LL_10_-ZnP_10_ blend, due to the formation of a protective char layer in the condensed phase that can effectively delay the heat release during combustion. LL contains sulfonate compounds, which are likely to decompose during combustion, releasing SO_2_, thus limiting the heat release and originating thermally stable Na_2_SO_4_ in the condensed phase [33,39]. It is noteworthy that the LL and ZnP formulation used in this study showed the enhanced fire performance compared to the cone calorimeter results presented in our previous study with lignin and ZnP combination [23]. When ZnP is replaced with AlP, PHRR remarkably decreases up to 230 kW/m² for PA_80_-LL_10_-AlP_10_. In addition, HRR curve reveals a broad and single peak of HRR, hence indicating the formation of an effective protective char layer. This superior fire-retardant property in forced combustion tests is due to the presence of an efficient aluminum phosphate layer, able to confer stability to the char residue. The increased char residue at the end of the test (Table 4) further confirms the formation of the protective layer. 

Furthermore, Figure 8 shows the influence of DL and ZnP/AlP on HRR and THR. It is worthy to note that, unlike LL lignin, the addition of DL with ZnP does not show a significant reduction in PHRR and THR. However, a certain reduction in PHRR (−43%) is seen when DL content achieves 10 wt %. Besides, the PA–DL–AlP blends show a remarkable reduction of PHRR (−64%) and THR (−22%) for PA_80_-DL_10_-AlP_10_. This is attributed to the presence of AlP, which promotes the formation of a stable char layer acting as a protective barrier, limiting the heat and mass transfer from and to the underlying polymer. 

Flame retardant action can also be confirmed by the effective heat combustion (EHC), which is expressed by the ratio of heat release to mass loss during combustion. EHC reflects the combustion efficiency of flammable volatiles. An obvious gas phase flame retardant action would lead to a noticeable reduction in EHC value as compared to the neat material [40]. Data collected in Table 4 show EHC for PA11 and the ternary blends, while the ternary blends containing 7 and 10 wt % of the lignins show lower EHC values; in this case, the flame inhibition occurs with an incomplete combustion, thus increasing the smoke and the CO release (Table 4) in the gas phase. 

### 3.5. Smoke and CO Release

The release of smoke and CO during combustion is not only a key fire hazard, but also an indication of the flame-retardant mechanism. For instance, a significant increase in the smoke release and CO amount is originated by incomplete oxidation of gaseous products, which indicates a flame inhibition action by radical trapping reactions taking place in the gas phase. Alternatively, a decrease in smoke release indicates a better-ventilated combustion process, in which the flame retardancy is dominated by fuel dilution and/or thermal barrier [35,41]. Therefore, total smoke release (TSR), CO and CO_2_ total yield, and CO_2_/CO yield were assessed; the corresponding data are collected in Table 4. First of all, the CO_2_ yield is practically similar for all the blends. As compared to PA11, binary blends with ZnP or AlP increase the smoke release and CO yield and lower the CO_2_/CO yield, hence indicating the incomplete oxidation of gaseous products. Conversely, the incorporation of LL and DL lowers TSR and CO yield and increase CO_2_/CO yield, also confirming the extended oxidation of the evolved gaseous products (Figure S4, Supporting information). 

**Figure 9:**
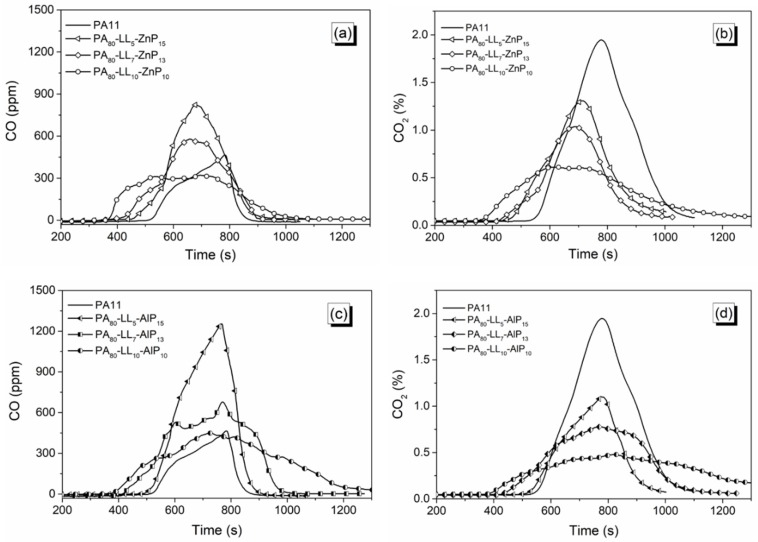
CO and CO_2_ evolution during combustion. (**a**) and (**b**) for PA–LL–ZnP blends; (**c**) and (**d**) for PA–LL–AlP blends.

On the other hand, the combination of lignin and phosphinate worsens the smoke parameters, confirming the occurrence of incomplete combustion. However, increasing the lignin content reduces the CO release, which is attributed to the more complete oxidation of evolved volatile products. Figure 9 shows the influence of LL mixed with flame retardants on CO and CO_2_ evolution. Interestingly, the blends containing 10 wt % of both fillers show the lowest CO release with respect to other ternary blends: more specifically, PA_80_–LL_10_–ZnP_10_ blend significantly lowers the peak of CO release up to 300 ppm without compromising the other fire-retardant properties. This finding can be ascribed to the interactions taking place between LL and ZnP in the ternary blend, which leads to extended oxidation of the evolved gaseous products as well as to the formation of a protective char in the condensed phase. Conversely, the peak of CO evolution reported in our previous study with lignin and ZnP combination showed a slightly higher, up to 500 ppm, for the same blend (i.e., 10 wt % concentration of both additives) [23]. Based on results, it can be concluded that the combination of lignin and phosphinate promotes the smoke and CO release; however, high lignin loading remarkably reduces CO release, hence minimize the smoke toxicity.

### 3.6. Morphology of Char Residue

**Figure 10:**
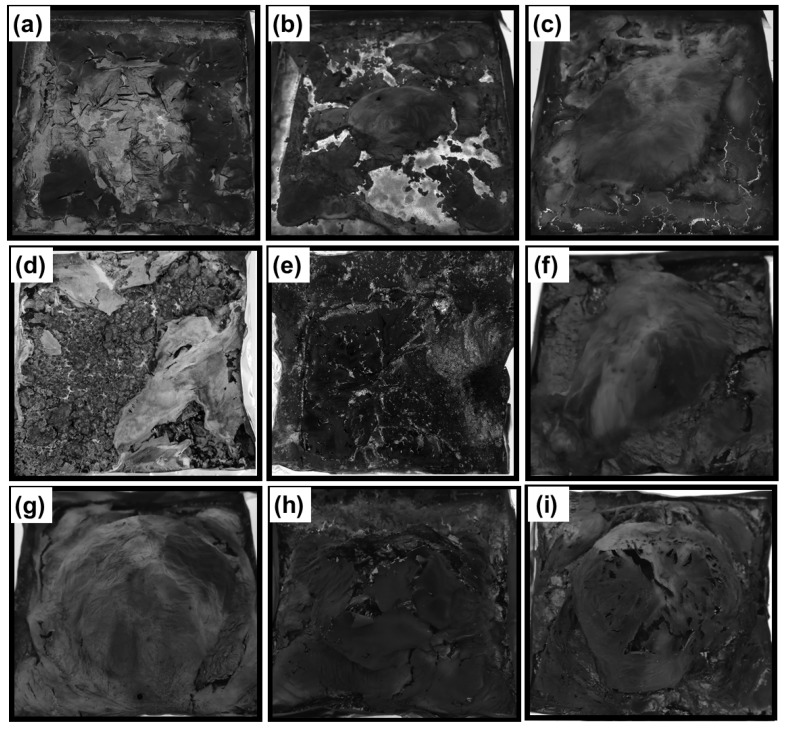
Pictures of char residues collected at the end of cone calorimetry test: (**a**) unfilled polyamide 11 (PA11); (**b**) PA_80_–ZnP_20_; (**c**) PA_80_–AlP_20_; (**d**) PA_80_–LL_20_; (**e**) PA_80_–DL_20_; (**f**) PA_80_–LL_10_–AlP_10_; (**g**) PA_80_–LL_10_–ZnP_10_; (**h**) PA_80_–DL_10_–AlP_10_ and (**i**) PA_80_–DL_10_–ZnP_10_ blends.

**Figure 11:**
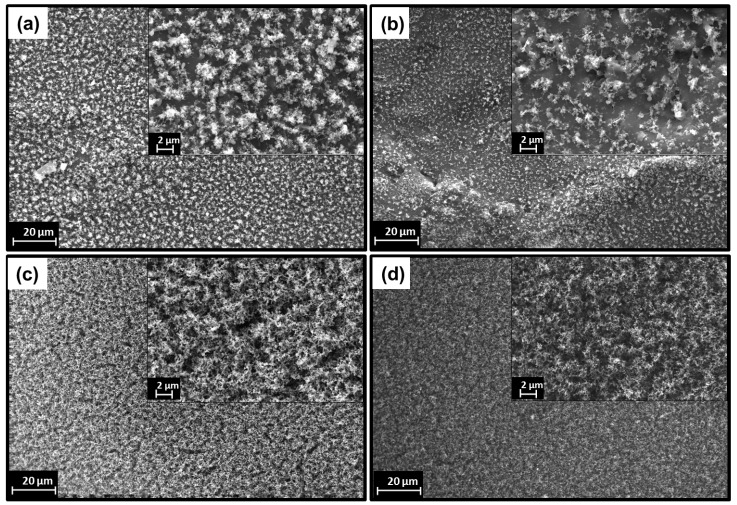
Scanning Electron Microscopy (SEM) micrographs of char residue surface after forced combustion for (**a**) PA_80_–LL_10_–AlP_10_; (**b**) PA_80_–LL_10_–ZnP_10_; (**c**) PA_80_–DL_10_–AlP_10_ and (**d**) PA_80_–DL_10_–ZnP_10_ blends at 1000× and 5000× magnification.

Figure 10 shows the pictures of residues obtained after forced combustion tests. It is clearly observed (Figure 10a) that the residue from PA11 is very thin and practically negligible. Similarly, PA11 with ZnP (Figure 10b) shows a mechanically thin and non-charring characteristic. However, PA_80_-AlP_20_ slightly increases the residue (Figure 10c), although the presence of cracks and insufficient residue leads to the formation of a weak protective layer. Besides, the binary blends of lignin (LL and DL) display a higher char residue formation: in particular, PA_80_-LL_20_ (Figure 10d) gives rise to a coherent and compact char layer formation, which protect the material against heat flux and reducing the release of flammable and non-flammable gases throughout the polymer. However, the presence of cracks deteriorates the performance. Similarly, the char residue of PA_80_-DL_20_ shows a loose and porous surface with many cracks (Figure 10e). Interestingly, the combination of lignin and phosphinate not only increases the char residue but also leads to the formation of a stable char layer with barrier features against heat flux and release of combustible gases. In particular, the addition of LL with ZnP/AlP (Figure 10f,g) shows a compact and protective layer due to sufficient char formation. Conversely, ternary blends of DL show a thin char layer and relatively loose structure with many cracks; more specifically, PA_80_-DL_10_-ZnP_10_ (Figure 10i) gives a fragile and mechanically weak char layer. In order to further investigate the microscopic morphology and structure of the charred layer, the top surface of char residues was observed by SEM (Figure 11a–d). It can be observed that the surface morphology of residue from PA_80_-LL_10_-AlP_10_ (Figure 11a) appears denser and more compact due to the increased char formation and interaction between LL and AlP leading to a stable protective char layer, which contributes to improved flame retardancy. Furthermore, EDX analysis reveals the presence of C, O, Na, Al, and P, which results in a more compact char residue. Similar char residue morphology with AlP is reported [42,43]. As shown in Figure 11b, PA_80_-LL_10_-ZnP_10_ presents a relatively less dense and compact structure in comparison with PA_80_-LL_10_-AlP_10_, although compact char layer is present with some holes on the surface. In contrast, the surface morphology of residue from DL containing blends (Figure 11c,d) does not form an intact char layer due to insufficient char formation during combustion; as a consequence, a loose and porous structure is formed. 

## 4. Conclusions

So as to advance the exploitation of industrial lignin, in this work, different lignins, i.e., LL and DL, were combined with phosphinate (ZnP and AlP) flame retardants in PA11. Different flame retarded blends were prepared using melt extrusion. The influence of the presence of lignin with phosphinate on the thermal and fire behavior was thoroughly assessed by TG analyses, UL 94 vertical flame spread, and cone calorimetry tests. SEM analyses of the blends showed that the addition of lignin and phosphinates up to 20 wt % minimizes the particle size due to the thermal mixing; in addition, EDX elemental mapping revealed a suitable dispersion of lignin and phosphinate within the polymer matrix. However, some small (<10 µm) agglomerates were observed in AlP containing blends. TG analyses showed that, regardless of its type, the incorporation of lignin in the ternary blends increased the thermal stability of PA11, promoting the obtainment of a stable char residue at the end of the tests. Through UL 94 vertical flame spread tests, it was possible to demonstrate that the combination of LL with ZnP effectively improves the flame-retardant properties by reducing total combustion time. More specifically, PA_80_–LL_10_–ZnP_10_ and PA_80_–LL_7_–ZnP_13_ achieved self-extinction and V-1 rating. Furthermore, in forced combustion test the interactions between lignin and phosphinate promoted a remarkable reduction of PHRR and THR. In particular, the best fire-retardant performance was found by combining LL with AlP (i.e., PA_80_–LL_10_–AlP_10_), resulting in a strong reduction of PHRR (−74%) and THR (−22%) values. On the other hand, smoke parameters, namely TSR and CO yield, increased in ternary blends; however, increasing the lignin loading effectively reduced the CO and CO_2_/CO yield compared to PA11. Particularly, PA_80_–LL_10_–ZnP_10_ blend shows minimum CO evolution without affecting flame retardancy. The morphology of char residue showed that the formation of compact char layer is primarily responsible for the improved flame-retardant properties. In conclusion, from an overall point of view, the direct use of industrial lignin with phosphinate in PA11 seems to be quite promising as far as fire retardancy is concerned, also considering the “biobased” character of both the polymer matrix and the lignins.

## Figures and Tables

**Table 1:** Polyamide 11 (PA11) blend formulations with different lignin and phosphinate.

Sample	Polyamide 11 (wt %)	Lignin (L ^1^ in wt %)	Phosphinate (P ^1^ in wt %)
PA11	100	-	-
PA_80_-L_20_	80	20	-
PA_80_-P_20_	80	-	20
PA_80_-L_5_-P_15_	80	5	15
PA_80_-L_7_-P_13_	80	7	13
PA_80_-L_10_-P_10_	80	10	10

^1^ Samples name are coded as PA_X_-L_Y_-P_Z_, where L is the kind of lignin, i.e., LL (lignosulphonate lignin from Domsjö Fabriker AB) or DL (kraft lignin from UPM Biochemicals), P is the type of phosphinate, i.e., ZnP (zinc phosphinate, OP950) or AlP (aluminium phosphinate, OP1230)

**Table 2:** Thermogravimetric data for polyamide 11 (PA11) and its blends in N_2_ and air.

Samples	*T*_5%_ (°C)	*T*_max_ (°C)	MMLR (%/min)	R_Exp_^1^ 700 °C (%)	R_Cal_^1^ (%)	*T*_max1_ (°C)	*T*_max2_ (°C)	MMLR (%/min)	R_Exp_^1^ 700 °C (%)	R_Cal_^1^ (%)
Atmosphere: Nitrogen						Atmosphere: Air				
PA11	396	423	2	1	-	454	574	1.3	2	-
PA_80_-LL_5_-ZnP_15_	373	472	2.4	5.8	6.7	464	553	1.8	7.6	12.5
PA_80_-LL_7_-ZnP_13_	364	467	2	8.2	7.5	462	543	2	6	12.3
PA_80_-LL_10_-ZnP_10_	326	465	1.8	12.7	8.6	465	559	2.2	6.8	12.1
PA_80_-LL_5_-AlP_15_	363	468	1.7	5.5	6.2	460	611	1.6	7.1	10.0
PA_80_-LL_7_-AlP_13_	349	470	1.6	7.6	7.1	472	610	1.8	5.9	10.2
PA_80_-LL_10_-AlP_10_	330	445	1.5	10.7	8.3	470	612	1.7	6.2	10.4
PA_80_-DL_5_-ZnP_15_	359	473	2	5.4	5.8	468	573	1.7	5.8	10.1
PA_80_-DL_7_-ZnP_13_	347	472	1.8	6.4	6.3	474	577	1.6	3.8	9.0
PA_80_-DL_10_-ZnP_10_	339	453	1.6	8.9	6.9	462	564	1.7	4.1	7.4
PA_80_-DL_5_-AlP_15_	334	469	1.7	5.7	5.4	458	601	2.2	7.3	7.6
PA_80_-DL_7_-AlP_13_	325	470	1.6	7.4	5.9	455	604	1.7	6.8	6.9
PA_80_-DL_10_-AlP_10_	313	457	1.5	7.8	6.6	455	583	1.6	5.1	5.7

**^1^** R_Exp_ = experimental residue; R_Cal_ = calculated residue.

**Table 3:** UL94 vertical flame spread test data for polyamide 11 (PA11) and its blends.

Samples	1^st^ Flame t_1_ (s)	2^nd^ Flame t_2_ (s)	Combustion time (t_1_ + t_2_)	Cotton Ignition	Dripping	Rating
PA11	11 ± 1	7 ± 1	18 ± 1	Yes	Yes	V2
PA_80_-LL_5_-ZnP_15_	11 ± 1	3 ± 1	14 ± 1	Yes	Yes	V2
PA_80_-LL_7_-ZnP_13_	4 ± 1	3 ± 1	7 ± 1	No	Yes	V1
PA_80_-LL_10_-ZnP_10_	6 ± 1	3 ± 1	9 ± 1	No	Yes	V1
PA_80_-LL_5_-AlP_15_	16 ± 1	6 ± 2	22 ± 1	Yes	Yes	V2
PA_80_-LL_7_-AlP_13_	24 ± 2	4 ± 1	28 ± 2	Yes	Yes	V2
PA_80_-LL_10_-AlP_10_	8 ± 1	4 ± 1	12 ± 2	Yes	Yes	V2
PA_80_-DL_5_-ZnP_15_	10 ± 1	4 ± 1	14 ± 2	Yes	Yes	V2
PA_80_-DL_7_-ZnP_13_	11 ± 1	4 ± 1	15 ± 2	Yes	Yes	V2
PA_80_-DL_10_-ZnP_10_	7 ± 1	3 ± 1	10 ± 1	Yes	Yes	V2
PA_80_-DL_5_-AlP_15_	22 ± 2	5 ± 1	27 ± 2	Yes	Yes	V2
PA_80_-DL_7_-AlP_13_	19 ± 1	4 ± 1	23 ± 2	Yes	Yes	V2
PA_80_-DL_10_-AlP_10_	18 ± 1	3 ± 1	20 ± 1	Yes	Yes	V2

**Table 4:** Cone calorimetry data for polyamide 11 (PA11) and its blends.

Samples	TTI (s)	PHRR (kW/m^2^)	Reduction (%)	THR (MJ/m^2^)	EHC (kJ/g)	TSR (m^2^/m^2^)	CO Yield (g/kg)	CO_2_ Yield (kg/kg)	CO_2_/CO	Residue (%)
PA11	154 ± 3	884 ± 4	-	92 ± 4	33.8 ± 0.6	1033 ± 1	33 ± 1	2.6 ± 0.1	79	0.6 ± 0.1
PA_80_-LL_5_-ZnP_15_	112 ± 12	560 ± 40	37	79 ± 2	29.3 ± 0.6	1799 ± 48	88 ± 1	1.9 ± 0.1	22	5.9 ± 0.2
PA_80_-LL_7_-ZnP_13_	92 ± 9	443 ± 21	50	77 ± 4	28.8 ± 0.7	1652 ± 151	92 ± 1	2.1 ± 0.1	23	6.2 ± 0.2
PA_80_-LL_10_-ZnP_10_	86 ± 9	315 ± 11	64	73 ± 2	28.4 ± 0.4	1691 ± 26	72 ± 2	2.0 ± 0.1	28	8.5 ± 0.3
PA_80_-LL_5_-AlP_15_	142 ± 11	554 ± 33	37	78 ± 6	30.3 ± 1	1959 ± 28	98 ± 3	1.9 ± 0.1	19	5.8 ± 0.3
PA_80_-LL_7_-AlP_13_	124 ± 10	420 ± 27	52	77 ± 3	29.6 ± 0.3	2034 ± 37	85 ± 3	2.0 ± 0.1	24	7.1 ± 0.3
PA_80_-LL_10_-AlP_10_	108 ± 12	230 ± 14	74	72 ± 4	29.4 ± 0.4	1995 ± 18	71 ± 6	2.1 ± 4	30	11.5 ± 0.3
PA_80_-DL_5_-ZnP_15_	150 ± 18	740 ± 23	16	79 ± 2	29.6 ± 1.3	1720 ± 36	71 ± 3	2.2 ± 0.1	31	3.8 ± 0.2
PA_80_-DL_7_-ZnP_13_	128 ± 14	678 ± 36	23	77 ± 3	30.2 ± 0.4	1800 ± 88	73 ± 4	2.1 ± 0.1	29	5.2 ± 1.5
PA_80_-DL_10_-ZnP_10_	116 ± 13	500 ± 48	43	75 ± 7	30.3 ± 1.2	1745 ± 62	61 ± 6	2.1 ± 0.1	34	8.2 ± 0.5
PA_80_-DL_5_-AlP_15_	174 ± 14	424 ± 39	42	76 ± 4	28 ± 1	1819 ± 40	99 ± 2	2.0 ± 0.1	20	7.7 ± 0.5
PA_80_-DL_7_-AlP_13_	140 ± 12	406 ± 34	54	74 ± 5	31 ± 1	1929 ± 45	90 ± 3	2.0 ± 0.1	22	8.6 ± 0.5
PA_80_-DL_10_-AlP_10_	95 ± 7	320 ± 10	64	72 ± 1	29 ± 1.3	1967 ± 68	86 ± 5	2.1 ± 0.1	24	10.4 ± 0.5

## Supplementary Materials

The following are available online at http://www.mdpi.com/2073-4360/11/1/180/s1, Figure S1: SEM images showing particle size distribution of pristine material, (a) ZnP, (b) AlP, (c) DL, (d) LL. Figure S2. TG curves of unfilled PA11 and neat materials in N2 (a) and air (b), Figure S3. HRR and THR curves of PA11 and the binary blends, (a) and (b) PA80-LL20, (c) and (d) PA80-DL20, (e) and (f) PA80-ZnP20, (g) and (h) PA80-AlP20 blends, Figure S4. CO and CO2 evolution during combustion for PA11 and the binary blends, (a) and (b) PA80-LL20, (c) and (d) PA80-ZnP20, (e) and (f) PA80-AlP20 blends. Table S1: Thermogravimetric data for PA11 and its blends in N2 and air. Table S2. UL94 vertical flame spread test data for PA11 and the binary blends, Table S3. Cone calorimetry data for PA11 and its binary blends.
