# Supplementary material for: Valorization of Industrial Lignin as Biobased Carbon Source in Fire Retardant System for Polyamide 11 Blends
Source: Polymers (Basel). 2019 Jan 21;11(1):180. doi: 10.3390/polym11010180 (PMC6401932; doi:10.3390/polym11010180)
Supplement: Supplementary file 1 [file polymers-11-00180-s001.pdf]

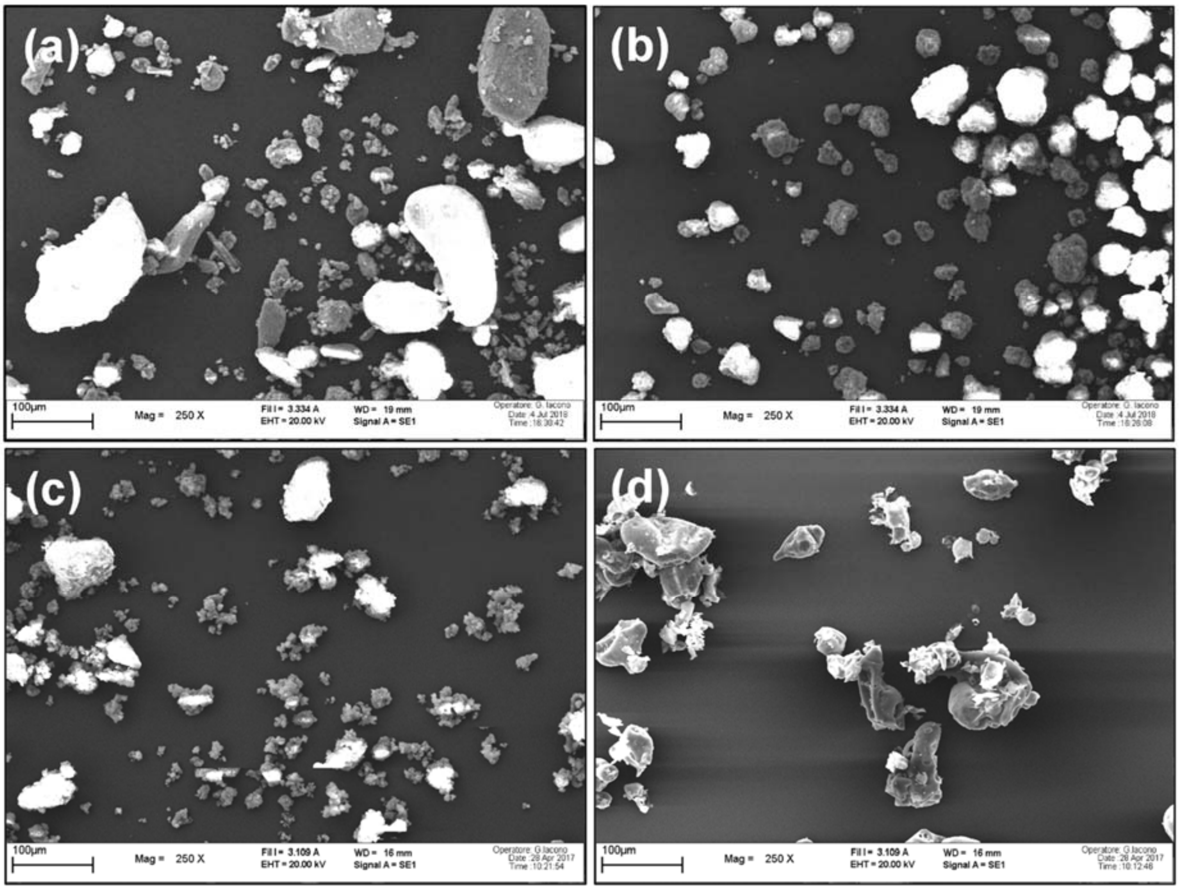

Figure S1. SEM images showing particle size distribution of pristine material, (a) ZnP, (b) AlP, (c) DL, (d) LL.

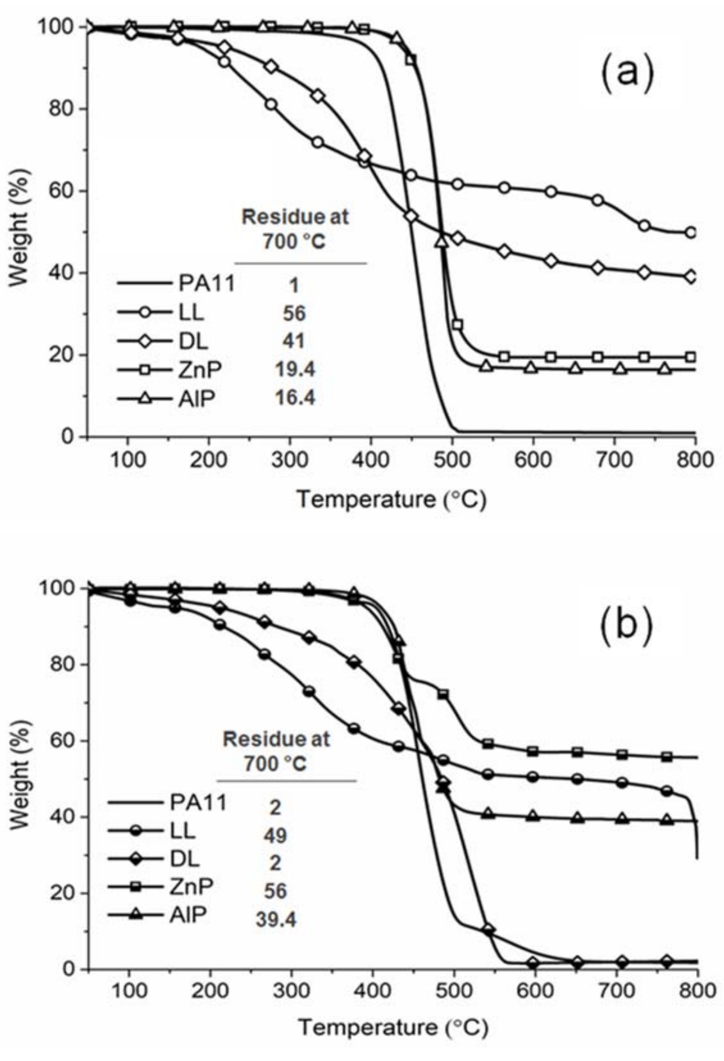

Figure S2. TG curves of unfilled PA11 and neat materials in N_2 (a) and air (b).

Table S1. Thermogravimetric data for PA11 and its blends in N_2 and air.

**Samples**	$T_{5\%}$ (°C)	T_{max} (°C)	**MMLR (%/min)**	R_{Exp}[1] 700 °C (%)	R_{Cal}[1] (%)	T_{max1} (°C)	T_{max2} (°C)	**MMLR (%/min)**	R_{Exp} 700 °C (%)	R_{Cal} (%)
**Atmosphere: Nitrogen**						**Atmosphere: Air**				
PA11	396	423	2	1	-	454	574	1.3	2	-
LL	189	249	0.22	56	-	337	515	0.22	49	-
DL	221	397	0.35	41	-	520	-	0.76	2	-
ZnP	437	485	1.8	19.4	-	428	509	0.47	56	-
AlP	442	485	2.6	16.4	-	432	476	0.86	39.4	-
PA_{80}-LL_{20}	285	468	1.6	13.5	12.5	469	585	2	5.3	11.4
PA_{80}-DL_{20}	341	435	1.4	12.4	9.1	442	587	1.5	2.5	2.0
PA_{80}-ZnP_{20}	366	473	2.3	1.2	4.8	457	537	2.2	7.5	12.8
PA_{80}-AlP_{20}	401	461	2.2	3.2	4.2	454	578	1.8	8.3	9.5

[1] R_{Exp} = experimental residue; R_{Cal} = calculated residue

Table S2. UL94 vertical flame spread test data for PA11 and the binary blends.

Samples	1st Flame t_1 (s)	2nd Flame t_2 (s)	Combustion time (t_1+t_2)	Cotton ignition	Dripping	Rating
PA11	11 ± 1	7 ± 1	18 ± 1	Yes	Yes	V2
PA_{80}-LL_{20}	5 ± 1	5 ± 1	10 ± 1	Yes	Yes	V2
PA_{80}-DL_{20}	9 ± 4	9 ± 3	18 ± 7	Yes	Yes	V2
PA_{80}-AlP_{20}	0	2 ± 1	2 ± 1	No	No	V0
PA_{80}-ZnP_{20}	21 ± 1	3 ± 1	24 ± 1	Yes	Yes	V2

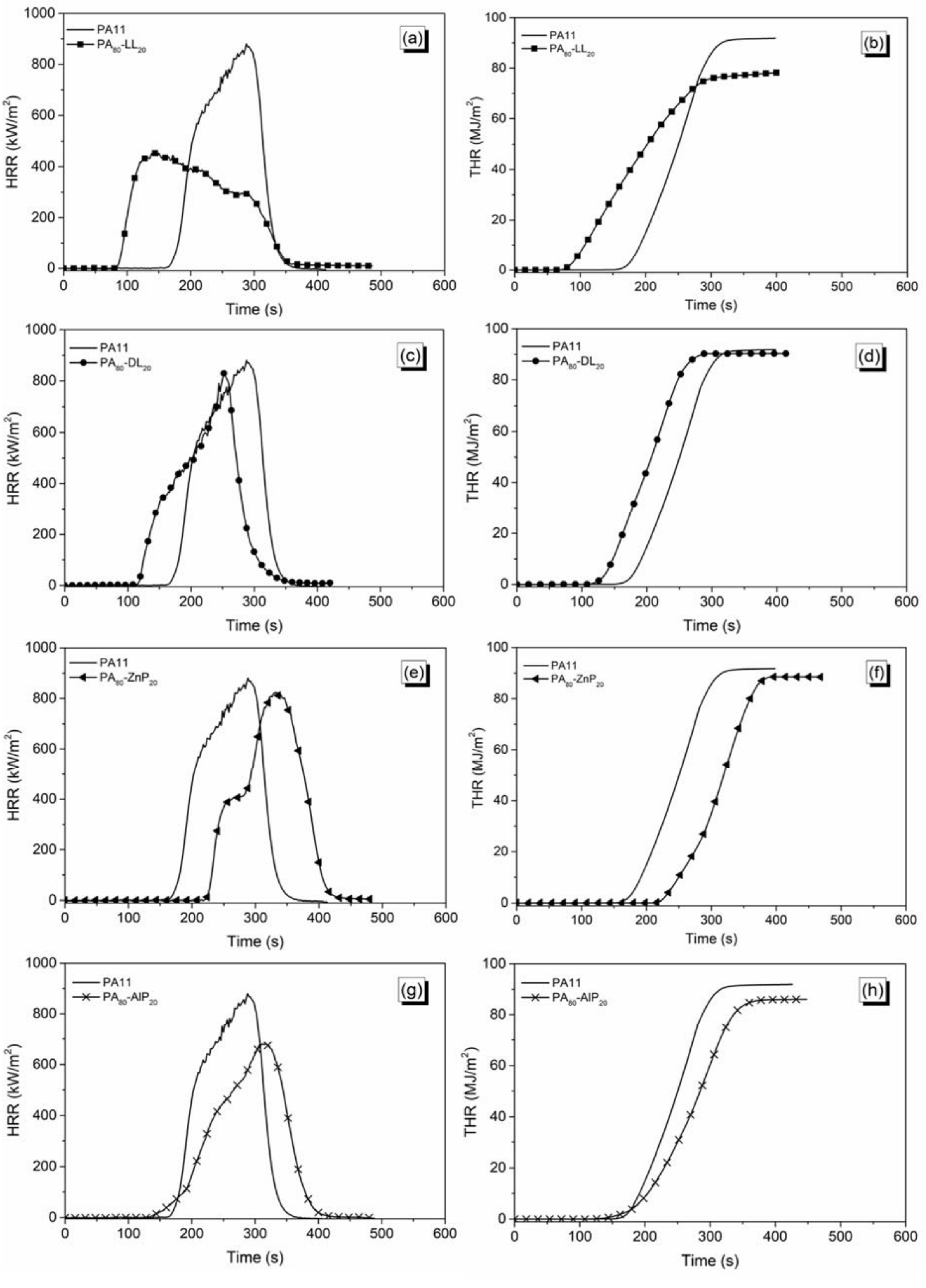

Figure S3. HRR and THR curves of PA11 and the binary blends, (a) and (b) PA_{80}-LL_{20}, (c) and (d) PA_{80}-DL_{20}, (e) and (f) PA_{80}-ZnP_{20}, (g) and (h) PA_{80}-AlP_{20} blends.

Table S3. Cone calorimetry data for PA11 and its binary blends.

Samples	TTI (s)	PHRR (kW/m²)	Reduction (%)	THR (MJ/m²)	EHC (kJ/g)	TSR (m²/m²)	CO yield (g/kg)	CO_2 yield (kg/kg)	CO_2/CO	Residue (%)
PA11	154 ± 3	884 ± 4	-	92 ± 4	33.8 ± 0.6	1033 ± 1	33 ± 1	2.6 ± 0.1	79	0.6 ± 0.1
PA_{80}-LL_{20}	72 ± 12	454 ± 30	49	78 ± 6	30.4 ± 0.5	1198 ± 20	29 ± 1	2.1 ± 0.2	72	8.7 ± 0.3
PA_{80}-DL_{20}	112 ± 10	821 ± 27	7	90 ± 2	30.1 ± 0.4	1290 ± 25	23 ± 1	1.6 ± 0.1	70	7.6 ± 0.1
PA_{80}-ZnP_{20}	223 ± 14	825 ± 29	7	88 ± 6	33.7 ± 0.5	1640 ± 62	94 ± 2	2.3 ± 0.1	25	1.4 ± 0.2
PA_{80}-AlP_{20}	114 ± 4	700 ± 33	21	86 ± 2	32 ± 1	2118 ± 41	164 ± 2	2.1 ± 0.1	13	4.2 ± 0.1

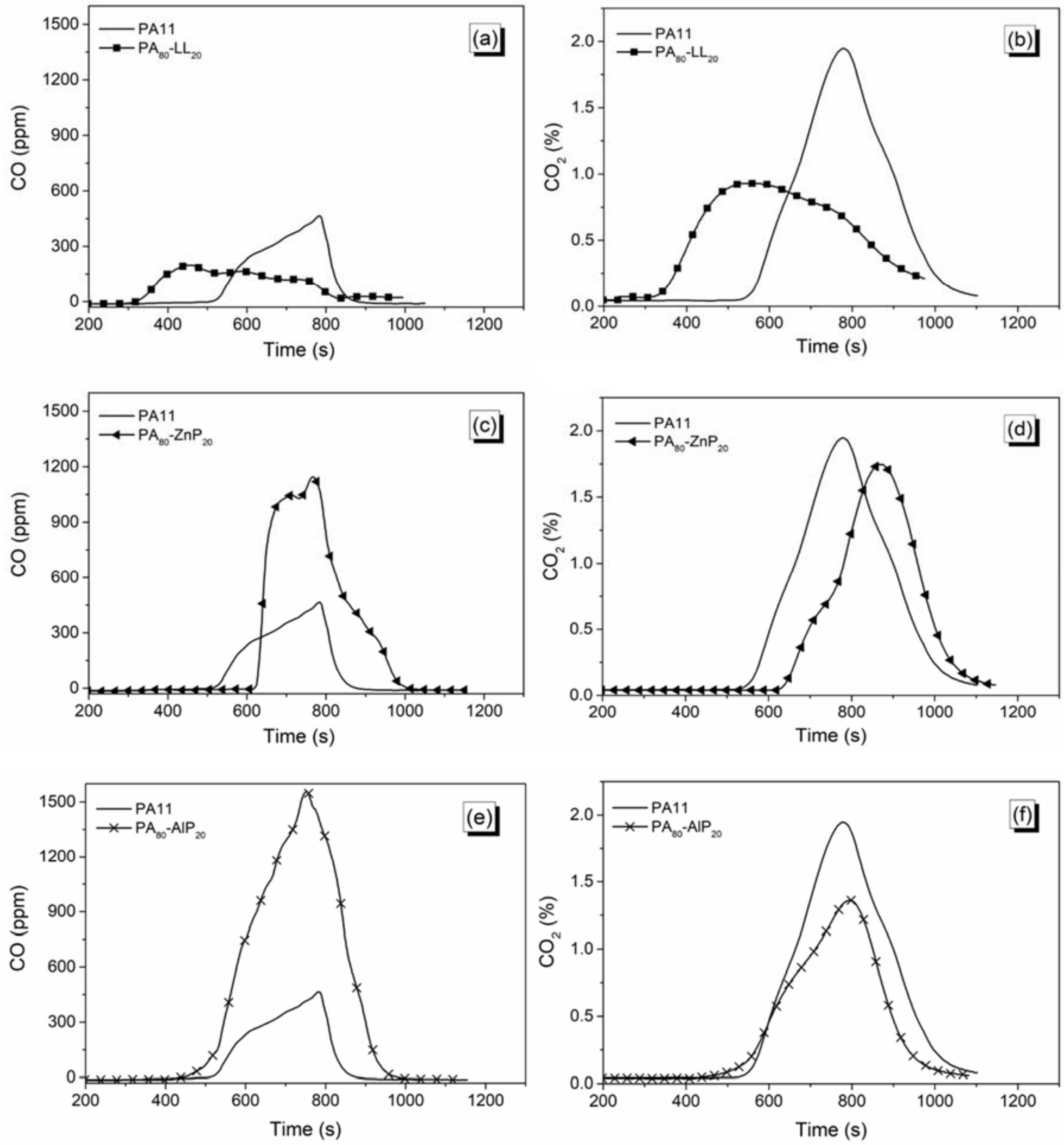

Figure S4. CO and CO_2 evolution during combustion for PA11 and the binary blends, (a) and (b) PA_{80}-LL_{20}, (c) and (d) PA_{80}-ZnP_{20}, (e) and (f) PA_{80}-AlP_{20} blends.
